# Concept Mapping of Career Motivation of Women With Higher Education

**DOI:** 10.3389/fpsyg.2020.01073

**Published:** 2020-05-27

**Authors:** Min Sun Kim

**Affiliations:** Department of Psychology and Psychotherapy, Dankook University, Cheonan, South Korea

**Keywords:** career, motivations, highly educated married women, concept mapping method, Korea

## Abstract

The purpose of the present study was to identify and categorize career motivations in highly educated married Korean women. Twenty-five participants who are working were interviewed and asked why they continue their work despite various difficulties. Sixty-seven career persistence motivations were elicited and reliably organized into six categories: low interest in childcare and household labor, family-related motives, high need for achievement, financial problems/needs, self-actualization and job satisfaction, and positive perception of working women. This study is significant as it conceptualizes the motivation for career persistence as the major variable affecting highly educated married women’s decision to continue their careers and establishes the theoretical basis for future research.

## Introduction

In many countries, women’s socioeconomic status has dramatically changed because of increasing access to education, full-time employment opportunities, and increased personal earnings relative to those of men (OECD, 2017). In South Korea, similar changes have occurred. Since the economic development of the 1960s, the share of women at four-year universities in Korea increased from 25.3% in 1970 to 73.5% in 2016 (Statistics Korea, 2016). As a result of the increased educational levels, the expansion of working opportunities for women has increased and many of them try to achieve professional success (Yoon, 2007).

Despite these advancements for women, household labor and childcare are still considered women’s primary role in Korea, and the belief that married women should be caregivers of their husbands and children is widespread (Jang and Merriam, 2005; Kang, 2011; Um and Yang, 2011). Because of the difficulty in maintaining careers and families, Korean women have many difficulties in balancing work and family and the proportions of Korean women who limit their childbearing to one child or avoid marriage or childbirth altogether is increasing (Kim et al., 2010; Choi, 2016; Choi et al., 2016). Also, the difficulties of balancing work and family obligations have been related to career disruption, job quality, gender wage gaps, and women’s status in the workplace (Kim, 2014). For example, a 2013 study found that the wage gap between Korean men and women was 35% (Kochhar et al., 2016) compared with 10% for the Organization for Economic Cooperation and Development Countries, and just 5% in northern European countries. The mean gender wage gap in northern European countries is in the five percentage point range (Kochhar et al., 2016). In addition, South Korea reported the lowest percentage of female managers among the OECD countries (10.5 v. 31.2%; OECD, 2017). Korean employers tend to lack systems tailored to meet women’s needs at various stages of the life course, and married women’s dual roles were limiting their opportunities for promotion, training, and development and that they had low self-efficacy, experienced job burnout, and reported depression in their career progress (Jang, 2008; Kang, 2011; Yoo et al., 2016). As such, women’s career interruption^1^ can lead to social problems such as social disparities or weak national competitiveness by avoiding marriage and childbirth and deepening the gap between men and women in society. Because women’s involvement in the workforce is vital to societal well-being, close attention should be paid to the difficulties faced by Korean married women in terms of their career persistence.

The conditions faced by women entering the labor market and their expectations regarding employment change as their educational attainment increases. However, highly educated women have reported that their jobs did not reflect their educational attainment and that their positions were less likely than men’s to lead to career advancement (Korea Research Institute for Vocational Education Training, 2018). Previous studies have shown that women consider the quality of work such as the job’s compatibility with the woman’s educational field, aptitudes, job demands, stability, and work conditions (Lee and Lee, 2010; Ministry of Gender Equality and Family, 2013; Choi, 2014). Some studies have found that highly educated married women are less worried about money and tend to pass up job opportunities that are not aligned with the ideal job (Kim, 2016). The spouses of highly educated women also are likely to be highly educated and to have stable jobs, so those women were found to quit their work when the opportunity costs are high (Choi, 2014; Lee, 2014). These results suggest that even if highly educated women restart work after career interruption, they are hard to continue and that their careers may be completely discontinued involuntarily. This study included only highly educated women who obtained degrees equivalent to or higher than a bachelor’s degree as the subjects, assuming that career patterns of married women may be different according to their academic background.

Some research has identified the role of married women’s career motivation as key elements in their career persistence (Zusho et al., 2003; Ratelle et al., 2005). However, there is still insufficient research on psychological factors affecting the career persistence of women including motivations. The lack of attention to the psychological characteristics that allow highly educated women to continue their careers is also linked to the social perspective of women. There is a prevailing perception in South Korea that married women value work only for money to ease financial hardships and difficulties. That assumption reflects a lack of investigation on the value and meaning of work in women’s lives. Assuming women’s motivations for work are purely financial negatively influences their persistence (Choi and Kim, 2007; Lee and Kim, 2010; Lee and Lee, 2010). But unlike these assumptions, the numbers of women who want to work for personal success, self-realization, and stable futures have increased across cohorts in South Korea (Jang and Bu, 2003; Hong and Lee, 2014; Ju and Joo, 2017). Also, married women who lacked a sense of professional identity or motives for their job choices considered quitting when they experienced workplace difficulties or needed to be primary family caregivers (Kim and Seo, 2015). Some previous studies found that women who were highly motivated for self-development or by the joy they derived from their work and identity as professionals were relatively likely to want to persist or be reemployed after a break (Choi and Kim, 2007; Kim, 2007; Jeon and Ju, 2016; Ju and Joo, 2017).

The previously conducted research mentioned above all examined the motivations among highly educated married women; however, these studies had limitations. First, most studies focused on highly educated women who are seeking reemployment or who have taken career interruption (Jang and Merriam, 2004; Kim, 2007; Yoon, 2007; Jang, 2008; Cho, 2009; Jeon, 2010; Um, 2010); thus, empirical research is lacking for women who are continuing in their careers without interruption. Second, since some qualitative studies (Cho, 2009; Jeon, 2010) revealed variables that can be considered as motivation for persisting a career, there were also limitations in defining major variables in explaining the process of women keeping their careers. Furthermore, some studies have only addressed individuals working in specific sectors, such as police officers, nurses, and teachers (Lee, 2003; Jung, 2012). Most of the existing studies focus on reemployment of women who have been discontinued, and there is a limit to examine the factors of career continuity for women in specific occupational groups. Intervention after career interruption has limitations in ensuring the quality of women’s jobs and the connection with previous jobs, and eventually many highly educated women are involuntary career interruption. Recent research have indicated that many highly educated women, who realize the negative impact of career interruption, try to maintain their careers even after pregnancy and childbirth (Becker, 1991; Desai and Waite, 1991; Yu, 2005; Goldin, 2006). Therefore, we aim to explore career persistence motivations focusing on cases of highly educated married women. This study will help women to share their career motivations according to cultural differences and to understand the career motivations of women from countries with similar social development and cultural backgrounds like Korea.

## Literature Review

Career motivations refer to the type, direction, strength, and maintenance of energy expended by individuals to obtain certain work-related outcomes (Pinder, 1998). Self-determination theory (SDT) (Deci and Ryan, 2000) comprehensively explains environments, motives, psychological satisfaction, and goal setting. Therefore, SDT is a good theoretical framework for explaining women’s career persistence, performance, and satisfaction. Kasser and Ryan (1993, 1996) categorized self-determinative (internal) motivations as self-realization, meaningful opportunities for self-improvement through work, development of meaningful or satisfying relationships with colleagues, and opportunities to help others. In contrast, honor, social status, and high salary are external motives for working. In the study of Bezzina et al. (2013), which examined women’s career motivation, 16 major motivations were derived: technology/ability utilization, success/achievement, vocational development, creativity, awareness, self-esteem, responsibility, social class improvement, economic independence, meeting people or making new friends, living expenses for basic life, and economic stability. This result means that married women have motivations reflecting various life-developmental characteristics and that there is a limit to understand the career continuity motivation of married women in terms of intrinsic/external motivation mentioned in the existing motivation theory.

Previous research found that self-determinative motives positively related to achievement and well-being at work (Baard et al., 2004), job satisfaction (Harter et al., 2002; Richer et al., 2002), job performance (Kuvaas, 2009), and commitment (Gagné et al., 2008). Other studies found negative associations with depression (Blais et al., 1993), burnout (Houkes et al., 2001; Fernet et al., 2004), physical ailments (Otis and Pelletier, 2005), and turnover intentions (Harter et al., 2002; Richer et al., 2002; Milette and Gagné, 2008).

Also, the interrelationships among career motivations, career persistence, and aspirations for professional success have been found in studies of South Korean married women (Sung and Ok, 2006; Choi and Kim, 2007; Jang, 2008; Kim, 2007; Son, 2013). Choi and Kim (2007) and Son (2013) found that working married women had high levels of motivation for professional accomplishments and clear goals. These women perceived their occupations as more than a way to earn money, and they considered their jobs as ways to fulfill social roles, express their unique abilities, and reach self-actualization. Previous research that compared women who continue to work and those who do not revealed that the former have a high motivation for professional accomplishments and clear goals. On the other hand, those who do not work appear to have low levels of motivation as well as ambiguity in their career goals (Choi and Kim, 2007; Jeon, 2010; Um, 2010).

### South Korean Women’s Work

Deci and Ryan (2000) noted that women’s motives might differ by sociocultural context. In Korea, feminism has been instrumental to changing women’s roles during the past two decades, and South Korean women have embraced the ideas of equal gender rights, women’s educational opportunity, social participation, and feminist family values (Choi, 2017). South Korean women are developing new social roles with progressive and active attitudes, improved education, and increased social participation. However, many of people still believe that household labor and childcare is women’s work. Won (2014) examined the changes in gender-role stereotypes in Korea from 1996 to 2010. The results of this study illustrated that traditional gender perception was eased in providing equal educational opportunities such as entering a college; however, rigid gender perception remained in job allocation in the labor market. In particular, the stereotyped evaluation of the mother’s role revealed that it was more strengthened in 2010 than in 1996. Despite the increase in the number of dual-income households, the fact that the awareness of traditional motherhood has been strengthened points to the difficulties that are faced by married women who participated in the labor market.

One of the environmental factors that affect the persistence of married women’s careers is the gender stereotypes that society has. It is true that policies for women are expanding at the national and corporate level, but in Korean society, prejudices about the roles of women and men shared by members and perceptions about women’s social activities are still rigid. Gender stereotypes are defined as “a set of structured ideas about the personal attributes of men and women” (Ashmore and Tumia, 1980, p. 502; Ashmore et al., 1986, p. 121). The prejudice based on gender stereotypes is based on the following logic (Bem, 1993). First, male-centeredness defines male experience as a norm and other experiences as “strange” beyond the standard, presupposing the superiority of masculinity. Second, there is a difference between masculinity and femininity as biological fundamentalism, and this difference is considered biological and essential. According to this logic, the dichotomy between the roles of men and women is natural, and when it is out of it, it is defined as unnatural and abnormal. In other words, it is unnatural and strange that women play expected roles in men or men play expected roles in women (Johnston and Swanson, 2006).

In particular, Korean society has a strong patriarchal characteristic centered on Confucian culture, and there is a clear boundary between the public area represented by the labor market and the private area represented by the family (Won, 2014). The former is defined as the male domain and the latter as the female domain. In Korean society, men are responsible for the livelihood of their families in the public sphere represented by the labor market, and women perceive that it is normal to fulfill the responsibilities of care for their families at home. Even if women have entered the public sphere due to social changes, this is an “inappropriate” position for women, so it has expanded to the logic that the resources and status in the public sphere should be more distributed to men who occupy the original sphere (Crompton, 2001; Lewis, 2001).

Gender-role stereotypes for women expand to create stereotypes for mother roles (Geist, 2005; Del Boca et al., 2009). Women enter the labor market, causing a gap in the role of mothers in the home, and the absence of mothers develops into the perception that they are negative for the emotional development and well-being of infants and toddlers, which leads to guilt for working women (Elvin-Nowak and Thomsson, 2001). Among surveyed countries, South Korea was the only one where men spent less than 1 h (45 min) per day on average performing household chores, which was much less than the average of 138 min (OECD, 2017). South Korean women spent 227 min on household labor per day, which was more than five times that of the South Korean men in the study (OECD, 2017). Other recent research has found that many married Korean women considered quitting their jobs because of the burdens of childcare and their children’s education (Yoo et al., 2014; Statistics Korea, 2015). Women tended to feel guilty for not properly behaving as a mother (Yang and Shin, 2011; Kim and Park, 2012; Jung and Kim, 2015), suggesting that the expected traditional family roles for women are dominant.

On the other hand, stereotypes about mother roles directly affect women’s perception of work and promotion in the workplace. In the study by Powell et al. (2002), men more actively agreed that women with young children should stay at home and that men should be the main source of income for households. Male managers placed female workers in step or support departments rather than key management positions to minimize the impact of family responsibility on work performance based on the perception of women and women’s roles. The study by Joo (2005), on the subject of female managers, also alludes to the male-centered culture and perception of female managers in the organization. In the Korean corporate culture, which is male-centered, it is necessary to adapt to the work-oriented culture created by men in order to succeed, and it is evaluated that there are accidental events and external factors rather than internal attributions such as work ability for women’s success. In addition, most leaders were male, so they felt uneasy about women becoming leaders of the organization, and they felt uncomfortable interacting with female bosses.

### Overview of the Present Study

The purpose of this research was to examine the concept and component factors of the career persistence motivations of married women in Korea using concept mapping. Based on these previous studies, this study assumed that highly educated married women would have various career persistence motivations. Especially, this career persistence motivations will reflect the cultural and environment context of women, and it is assumed that the unique motivations of Korean women will be derived.

The concept mapping process enables participants to express their perception through structured interviews and quantifies the results thereof (Kane and Trochim, 2007). Thus, the concept mapping method is an appropriate approach to explore the underlying structures of perception. It shows various ideas (statements) at once and how close they are on a concept map. In this study, the following steps of the concept mapping method were followed to collect and analyze the data. The data collection and analysis procedures are presented in the order of the following steps, which are (a) formulating research questions, (b) conducting interviews, (c) creating statements through participant interviews, (d) structuring the statements into groups of conceptually similar statements, (e) rating the importance and similarity of the statements, (f) running two-dimensional multidimensional scaling (MDS) on the group-sort data, (g) running a hierarchical cluster analysis (HCA) (to decide on a final cluster solution), (h) labeling the clusters, and (i) interpreting the concept maps (Trochim, 1993; Trochim et al., 1994).

## Materials and Methods

### Participants

The sample consisted of 25 married working women aged between 28 and 48 years, with a mean of 34.80 years (*SD* = 4.93). This study referred to the suggestions of Trochim (1989) which introduced the concept mapping method in determining the number of participants. Twenty participants reported that they had obtained a bachelor’s degree, and five had obtained a master’s degree. At the time of the interviews, they had 2–25 years of work experience, with an average of about 11.60 years (*SD* = 5.51). The participants had at least one child who ranged in age from 1 to 20 years (*M* = 5.00, *SD* = 4.33). Specifically, 16 participants (64.0%) had one child, 8 (32.0%) had two children, and one (4.0%) had three children. In this study, 20 participants reported that they earned an annual salary of at least 30 million won. Four participants reported salaries between 20 and 30 million won and one participant reported a salary of 20 million or less.

The participants were recruited through (1) snowball sampling and (2) online community advertisements. To be included in this study, participants had to meet the following criteria: (1) married women who have full time job; (2) married women who have no more than 1 year of career interruption, even during pregnancy and childbirth; (3) married women who have more than one child; (4) married women who aged under 45 years; and (5) married women who have earned at least a bachelor’s degree. Then, the principal investigator conducted telephone interviews for selecting participations, after which 25 of the 40 potential participants who met the inclusion criteria were selected for the final analysis.

### Data Collection

The concept mapping method (Trochim, 1989) used in this research is based on late positivism (Wheeldon, 2010). While it uses objective and standardized procedures, it is based on the constructivist notion that social reality is relative (Kane and Trochim, 2007). The concept mapping method collects data through qualitative approaches to derive subjective experience of the research subject. However, it includes both quantitative and qualitative research in that it verifies the collected data using quantitative methods (Daughtry and Kunkel, 1993; Paulson et al., 1999; White and Farrell, 2001; Kane and Trochim, 2007). So far, there have been few attempts to understand the characteristics and concepts of highly educated married women’s motivations to continue their career. Therefore, this study used the concept mapping method (Trochim, 1989) to determine how highly educated married women perceive such motivation. The questions asked in the interviews were formulated based on the literature review and procedures suggested for the concept mapping method.

The interviews were conducted face-to-face (*n* = 25). During the face-to-face interviews, which lasted from 50 to 60 min, participants were asked to freely speak about their experiences regarding the focus question. After each interview, the principal investigator transcribed the recorded interviews, and extracted all statements that could be considered motivations. The principal investigator then mailed participants and asked them to compare the statements with what they had written at the end of the interview.

### Sorting Procedures and Data Analysis Strategy

The 25 participants generated 308 statements about career persistence motivation. After all interviews were completed, researcher(s) worked to pare down the 308 statements, and a 10-member team of interviewers examined the adequacy of the excluded statements. In total, 67 unique and clear statements were identified during the data reduction process. Following this, the principal investigator sent 67 cards with the statements and asked the 25 participants to perform two “structuring” tasks with the 67 statements (i.e., task-sorting cards and rating statements). First, they were asked to group cards with similar meanings using the following rules. Sorting rules were as follows: (1) all statements (cards) could not be put into a single group, (2) all statements (cards) could not be put into their own separate groups, (3) one statement (card) could not be put into two groups, and (4) there could not be any “miscellaneous” groups. Second, they were asked to rate each of the 67 statements of career persistence motivation in terms of importance and degree of similarity to their own experiences.

A HCA using Ward’s algorithm is a widely used method for dividing statements into clusters of similar concepts (Kane and Trochim, 2007). Specifically, HCA partitions the point map created through MDS into clusters of related statements and superimposes this cluster solution onto the two-dimensional MDS plot (Bedi and Alexander, 2009). The cluster boundaries around groups of points represent statements that are more frequently sorted together. The key task is to decide on the number of clusters. For this, the researcher examined which cluster solution made sense theoretically, and then made a final decision on the number of clusters. Following this, the researcher examined the overall statements distributed on both sides of the *X* and *Y* axes to determine whether they served their intended purpose as dimensions in explaining the characteristics of each statement. All concept mapping data were analyzed using SPSS 18.0.

## Results

### Multidimensional Scaling Analysis

A 67 × 67 binary square matrix was created from the responses of the 25 participants. Values in the matrix were either 0 or 1, where 1 indicated that the two statements were placed in the same pile. The next step was to sum the similarity ratings of the 25 participants. From that matrix and through the MDS, a two-dimensional concept map of distances showing the similarity of statements between the 67 statements based on the sorts of the 25 participants was created.

Stress values measure the degree to which the distances on the map are discrepant from the values in the input similarity matrix (Kane and Trochim, 2007). In this study, two-dimensional solution was selected based on stress values (stress value = 0.13). Kruskal and Wish (1978) indicate that it is generally easier to work with two-dimensional configurations than with those involving more dimensions, and that ease of use considerations are also important in decisions regarding dimensionality. The literature on MDS (Kruskal and Wish, 1978) argues that it is desirable to have a stress value of 0.10 or lower. However, Clarke (1993) mentioned that if stress values are above 0.20, misinterpretation of the results can be derived, but less than 0.20 is an acceptable criterion. Also, Trochim (1993) noted that a stress value of 0.10 or lower is an inappropriate standard in most concept mapping contexts, and stress values in concept mapping research are likely to range between 0.20 and 0.36.

**FIGURE 1:**
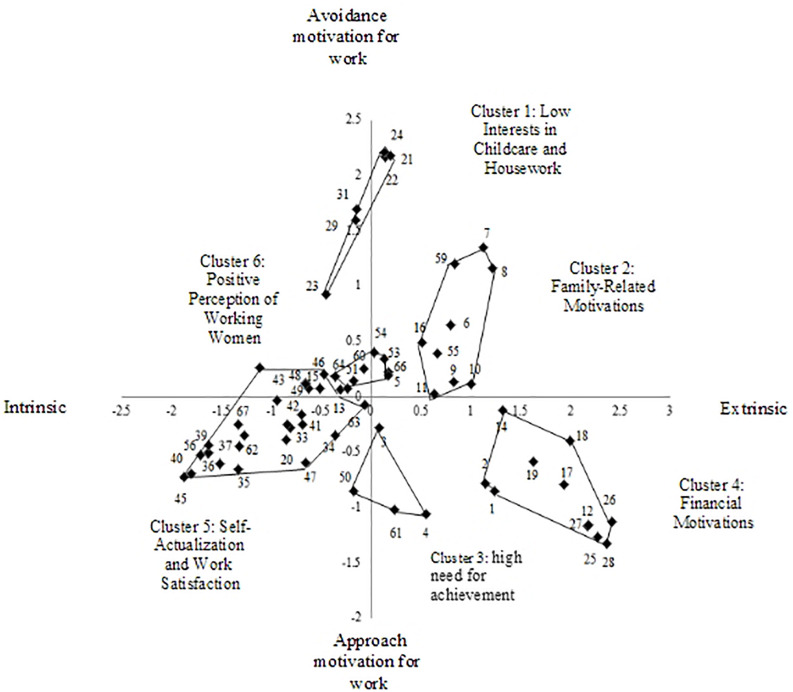
Cluster map of married women’s career persistence motivation.

The researcher examined the overall statements distributed on both sides of the *X* and *Y* axes (see Figure 1). First, statements that represented intrinsic motivation such as finding enjoyment in work or one’s identity as a working individual were located on the left side of the axis, while extrinsic motivation such as relational and economic factors emerged on the right side of the axis. Regarding the reasons for keeping their careers, highly educated married women perceive the intrinsic motivation as related to work itself, while extrinsic motivation includes relationships and financial factors. In other words, married women’s reasons for continuing to work reflect an intrinsic perspective such as the sense of accomplishment, satisfaction, and attachment associated with work itself, as well as an extrinsic perspective such as relationships and financial reasons. These reasons are similar to the intrinsic and extrinsic motivation discussed in the existing motivation research and theory (Deci, 1971, 1975; Clayton and Smith, 1987). In keeping with SDT (Deci and Ryan, 2000), these findings indicate that motives spring from different degrees of self-determination depending on the extent to which the motivation is internalized. As such, high self-determination indicates an internalized motive based on the personal meaning and importance of work. On the other hand, external factors stemming from low self-determination play an important role in women’s persistence to work and lead to different results from those of internalized motivation. Accordingly, the *X*-axis was labeled as “source of motivation” (intrinsic-extrinsic motivation for work).

Next, statements that represented approach motivations such as the high need for achievement and financial motivations were distributed at the bottom of the axis, while those that represented avoidance motivations such as avoiding childcare and housework were distributed at the top. An approach motivation refers to the actions taken to produce positive and ideal outcomes, while an avoidance motivation refers to actions taken to avoid negative or undesirable matters or possibilities (Elliot, 1999). In addition, avoidance motivation results in actions for survival, rather than in efforts to achieve a certain goal. Performance-avoidance and mastery-avoidance goals may lead to anxiety and negligence in the context of achievement and so lower performance and internal motives (Cury et al., 2006). Therefore, the *Y*-axis was labeled as “direction of behavior with regard to positive or negative stimuli” (approach-avoidance motivation for work).

### Hierarchical Cluster Analysis

In this study, two-stage clustering as suggested by Hair and Black (2000) was performed. First, the number of clusters was determined based on the variability and interpretation of the aggregation schedule developed through Ward’s hierarchical clustering method. Assuming four and five clusters, the difference in the coefficient dropped sharply, but started to slow down from six. In the second stage, the average score of each group was put into the seed points and the K-means analysis was performed (Hair and Black, 2000; Everitt et al., 2011).

The conceptual clusters in the two-dimensional stress map were as follows (see Table 1): low interest in childcare and housework (Cluster 1) (seven items, e.g., “I continue working because I am not interested in childcare and housework”), family-related reasons (Cluster 2) (nine items, e.g., “I continue working because I may have to participate more in my husband’s family affairs if I quit my job”), high need for achievement (Cluster 3) (six items, e.g., “I continue working because I am afraid of the severance of my working experience”), financial issues (Cluster 4) (nine items, e.g., “I continue working for financial reasons like education fees, purchasing a house, and living expenses”), self-actualization and work satisfaction (Cluster 5) (28 items, e.g., “I continue working because my work enables me to feel my existence, value, and identity”), and positive perception of working women (Cluster 6) (eight items, e.g., “I continue working because I want to become a good role model to the next generation as a new type of women, rather than playing a role as a traditional housewife and mother”).

**TABLE 1:** Statements by career persistence motivation cluster.

	**Statement (examples)**
Cluster 1	Low Interests in Childcare and Housework (Seven Items)
21	I continue working because I am not interested in childcare and housework.
22	I continue working because childcare and housework do not fit my aptitude.
24	I continue working because I get more stress from childcare than my work.
30	I continue working because my work becomes a hideout from housework and childcare.
29	I continue working because I feel kind of relaxed in my work as having more free time and getting out of childcare.
Cluster 2	Family-Related Motivations (Nine Items)
9	I continue working because I can understand better of my husband’s social life and I do not have to fully depend on my husband in our relationship.
10	I continue working because my children and husband might recognize and be proud of my work.
11	I continue working because I want to be a good role model to my children who have to keep working even after getting married in the future.
16	I continue working because I have been educated to continue working since I was young and my mother also wants me to keep working.
59	I continue working because my husband’s family understands me even when I cannot participate in family events or I am not good at housework.
Cluster 3	High Need for Achievement (Six Items)
4	I continue working because I am afraid of the severance of my work experiences.
61	I continue working because my competitiveness would drop in my area and also it would be tough to adapt to the rapidly changing environment when quitting my job.
50	I continue working because I do not want to give up my work experience built up in the current work.
3	I continue working because I am afraid of falling behind my friends if I quit my job.
1	I continue working because it would be impossible to get a job in the same condition as the one now after quitting this job.
Cluster 4:	Financial Motivations (Nine Items)
18	I continue working because I can give some help to my parents’ home when it is necessary without trying to read my husband’s mind.
19	I continue working because I can equally contribute to domestic economy and become confident with husband and his family.
14	I continue working because my husband wants a dual income as a requirement.
25	I continue working for financial issues like education fee, purchasing a house and living expenses.
26	I continue working because it would be tough to maintain the current lifestyle if I quit my job.
Cluster 5	Self-Actualization and Work Satisfaction (Twenty-eight Items)
34	I continue working because I think that I should not give up my work to live independent life without depending on others.
47	I continue working because I am accustomed to my stable job as my work experiences get longer.
20	I continue working because I am scared of losing my own identity and something that can explain myself when quitting my job.
35	I continue working because my work enables me to feel my existence, value and identity.
44	I continue working because I am satisfied with the current job and work.
Cluster 6	Positive Perception of Working Women (Eight Items)
5	I continue working because I feel guilty about leaving the company for personal reasons like childcare.
60	I continue working because I might have narrow views to the world and feel degraded as a woman and a member of society when concentrating on only childcare.
64	I continue working because I want to become a good role model to the next generation as a new type of women, rather than playing a role as a traditional housewife and mother.
51	I continue working because I do not understand quitting my job due to marriage and childcare as continuing work after getting married has been such a natural thing since I was young.
52	I continue working due to the responsibility for my work in the company.

### Importance and Experience Similarity Ratings

The average rating of the importance and similarity of each cluster, as understood by participants, was calculated by taking the average rating of importance attached to the statements (see Table 2). An examination of the cluster importance shows that the “Financial Motivations” was the most important cluster (important rating average = 3.80). “Positive Perception of Working Women” had the lowest cluster average importance rating of all the clusters (important rating average = 2.57). Next, the average rating of similarity to participant’s own experiences was calculated by taking the average rating of similarity that provided to the statements. Participants rated the “Financial Motivations” as the most similar cluster with their own experiences (similarity rating average = 3.68. Participants rated the “Low Interests in Childcare and Housework” and “Positive Perception of Working Women” as the lowest similar cluster with their own experiences (similarity rating average = 2.50).

**TABLE 2:** Importance and similarity rating average.

**Cluster name**	***M***	***SD***	
Importance Rating	Cluster 1 Low Interests in Childcare and Housework	2.65	0.81
	Cluster 2 Family-Related Reasons	3.14	0.65
	Cluster3 High Need for Achievement	3.60	0.67
	Cluster 4 Financial Issues	3.80	0.72
	Cluster 5 Self-Actualization and Work Satisfaction	3.70	0.62
	Cluster 6 Positive Perception of Working Women	2.57	0.57
Similarity Rating	Cluster 1 Low Interests in Childcare and Housework	2.50	0.99
	Cluster 2 Family-Related Reasons	3.04	0.76
	Cluster 3 High Need for Achievement	3.50	0.81
	Cluster 4 Financial Issues	3.68	0.90
	Cluster 5 Self-Actualization and Work Satisfaction	3.54	0.65
	Cluster 6 Positive Perception of Working Women	2.50	0.74

*Note. Statements within each category were rated for importance and similarity on a seven-point Likert type scale ranging from 1 = not important/not similar to 7 = extremely important/extremely similar.*

## Discussion

The motivation of highly educated married women is grouped into the following clusters. In total, six career motivations were identified, including “low interest in childcare and housework,” “family-related reasons,” “high need for achievement,” “financial reasons,” “self-actualization and work satisfaction,” and “positive perception of working women.”

First, low interest in childcare and housework included motivations such as avoiding childcare and housework, participating less in housework or affairs relating to in-laws, and preparing for the time when children grow up and no longer require care. In Korea, a study by Jang (2002) revealed consistent results among married women concerning avoidance motivations stemming from childcare. In Jang’s study, attending university was a good justification for escaping forced childcare for married Korean women who reentered university. These results are further explained in an earlier study (Um and Yang, 2011) that revealed that highly educated women want to continue their work, because they are more interested and satisfied with it than with childcare and housework. Moreover, in Korea, if women do not work, they are expected to take full responsibility for childcare and household chores, and more actively participate in family events (Kim and Seo, 2012).

Second, highly educated married women in Korea appeared to consider issues pertaining to relationships with their husbands and parents-in-law as important motivators to retain their careers. Korean women are still keenly aware that as soon as they are married, they have to undergo the process of being accepted as a member of the family by sacrificing themselves for their husbands and parents-in-law. In a study on highly educated married women in India, Dubey and Tiwari (2014) found that a main motive was “because husbands want to continue working.” This means that in a collective culture, family expectations can be an incentive to continue working.

Next, the high need for achievement was also mentioned as a motivation for women to continue working. The items included fear of losing competitiveness in the field after giving up work or a desire for high achievement and competitiveness. In particular, women feared that they might have to opt for the types of jobs easy for them to obtain, rather than regaining employment in the field they worked in (Lee and Kim, 2014). These results suggest that women perceive high social obstacles and have low expectations in terms of being able to work again in the same field after giving up work for childcare or housework responsibilities at a point in life when they should be working (Kim, 2003; Ryu et al., 2016). The results of this study reflect these constraints, based on which Korean women are perceived. Therefore, it is necessary to review European cases including flexible working and working hours to help married women continue their work (Yang, 2018).

Fourth, financial issues were found to be a major factor in the work life of highly educated women in Korea. Examples of items included for the financial motivation factor, apart from responding to financial needs or difficulties, were financial support for children and financial freedom. In a study by Bezzina et al. (2013) on Belgian married women, economic stability was derived as a major motivation. In addition, the inclusion of the item “achieving financial independence” implies that financial independence is important for married women (Jang and Merriam, 2005; Lee and Lee, 2014). This research also replicates the findings of previous studies that indicated the importance of a multi-dimensional understanding of financial motivations, which have different characteristics. In particular, financial independence from a spouse and the desire to secure their own income were major financial motivations (Ismail, 2012).

Moreover, in line with the Korean sentiment that encourages parents to make sacrifices for their children’s education, many women continue working to earn money to cover private education expenses. In fact, economic motivation was the highest in the importance and similarity evaluation of experience, and aspects in this group are connected to the economic needs for family and child support, indicating the relationship-focused characteristics of Korean women. Therefore, it is necessary to understand the economic motivation of Korean married women in connection with their relational motivation. In Korea, children’s success tends to be perceived as the mother’s success; thus, women’s financial support for children’s education is gradually gaining importance, which is contrary to how thus far in South Korea, only the emotional role of women has been emphasized in their relationship with children (Kim and Siegfried, 2001). Consequently, highly educated married women may feel a double burden, as they must provide both emotional support as mothers and financial support, which may pressure them to maintain their careers involuntarily despite the difficulties experienced in having multiple roles.

Next, self-actualization and work satisfaction are frequently mentioned in previous research, as is enjoyment of the work and attachment to it. In particular, the fact that one’s identity can be explained through work was mentioned as a major motive for married women. For example, women perceived work as having an important role in identity formation, since work is regarded as self-development, whereas childcare and housework involve taking care of others. This result was consistent with that in prior research, and can be interpreted as the internalization of a value system that regards paid work as more valuable than childcare and housework (Lee and Lee, 2014). These results are similar to results from studies conducted in other countries, which showed that women continue their careers for personal growth (Faisal and Khan, 2011; Bezzina et al., 2013).

Furthermore, this research found that positive perception of working women provided another compelling reason for highly educated women to continue to work. They have a clear identity as working professionals, not as housewives, and do not consider giving up their careers for childcare and housework. In addition, they mentioned the benefit of providing role models for their children—male and female—in terms of working outside the home. Choi and Kim (2007) further revealed that women who continued to work had a higher level of self-acceptance as professional workers. Although these women experience similar stress from playing multiple roles in their lives, they display a higher motivation for self-fulfillment and expend more effort to achieve definite career goals.

As mentioned in the beginning of this study, sexual stereotype that exists in Korean society is commonly derived from clusters. In this study, the majority of participants had the greatest conflict with maternal roles, similar to those mentioned in the existing theory (Heiss, 1981) and studies (Elvin-Nowak and Thomsson, 2001; Del Boca et al., 2009), and the most difficult problem in continuing work was the problem of childcare. Women mentioned feeling guilty about not raising children directly, and most women had internalized social prejudices about their mother roles. This may reflect the reality of Korea, where the role of childcare and education is concentrated on women. Previous studies (Joo, 2005; Won, 2015) revealed that the higher the subjective satisfaction of the economic situation, the lower the negative impact of the mother’s participation in the labor market on the development of children, and the higher the satisfaction of the dual role performance as mothers and workers. In this study, it was found that participants with high salaries showed less conflict about quitting their jobs and the more alternatives to raise children were found. Also, the participants who received high salary expected that their children would be able to invest more for education and to play a better role as parents after their children became upper graders in elementary school. However, the participants with relatively low salary showed high conflicts between work and family. Therefore, it is necessary to discuss alternatives that can provide sufficient compensation by improving the quality of women’s jobs, and the fact that women in Korea undergo more conflict between child rearing and work may be linked to the problem of wage gap between men and women.

Finally, the following section examines the importance of the motivation of highly educated married women to maintain their careers and the degree of similarity to their own experiences. The participants rated financial motivations as the most important and perceived it to be most similar to the motivation they actually experienced. In addition, they rated the statement “I continue working for economic affluence in my family” as the most important and perceived it as most similar to the motivation they experienced. When most participants mentioned financial motivations during the interview, they focused on living a better life or securing the means to pay for their children’s education. The results reflect their investment and involvement in private education (Bae et al., 2015). In addition, a strong determination to achieve financial independence from their spouse was evident. This may be regarded as a reflection of women’s determination to be an equal, independent agent in the household through financial independence from the spouse. This is aligned to the increase in women’s tendency to explain their identity as an agent performing financial activities (Lee and Lee, 2014). This finding replicates the results of previous studies that confirmed that the financial needs (e.g., child education, family expanses) women experience in their life stages become important factors in motivating them to continue working (Jang, 2002; Rosada et al., 2013).

### Implications and Recommendations

Several implications regarding the improvement of social perceptions and design of support systems for highly educated married women may be drawn from this study. First, this research provides opportunities to think about the personal meanings that married women give to their work and to alter social perceptions of married women’s work. The women who participated in this study constantly experienced conflict between work and family as they continued working without career interruptions (several participations #1, #4, #10 stated “I think it was the hardest thing to have no one to take care of. I was thinking about quitting because I didn’t have time to take care of my children properly and I couldn’t concentrate on my work due to childcare.”). However, their identities in connection with work resulted in their high self-esteem and strong sense of positive perception of working women. They had the positive perception that, despite the difficulties of their present circumstances, they may be better role models as working mothers in the future. They perceived work and family as a single process of adapting to two different roles, rather than as independent areas that require them to base their identity in one over the other. Thus, these women demonstrated an internalized motive for work at the personal level and found much satisfaction in their work—they believed work to be a matter of personal choice.

In this study, women wanted to continue working for self-development and personal success, even after marriage. In particular, high need for achievement was assessed as the important motivation in assessing participants’ importance and experience similarity. In fact, many of the women who participated in the study were concerned that if they quit their jobs for childbirth or childcare, they would not be able to find work in line with their previous experience or would fall behind in terms of social achievement. These results reveal the necessity for flexible career development and management for women, as well as improvement of their expertise, based on their life cycle. Specifically, it is necessary to keep track of and manage the career development of female employees in all positions to manage the career interruption of talented female employees in companies. In addition, it should be stipulated that women are assigned tasks in which they can utilize experience from their previous career even after career interruption (participants # 15, #27 stated “Women who have experienced career interruptions in our country are forced to find mart cashiers or simple chores, but I think we need to further expand the system that allows women to work on their previous careers.”). It is also necessary to devise a plan for motivating women who consider their interests and expertise in their work as important beyond the work culture, career planning, and compensation system according to men’s standards.

This paper also has theoretical implication as follows. In this study, career motivation of highly educated married women was classified according to intrinsic-extrinsic criteria, which was similar to the assumptions of Ryan and Deci’s (2000) SDT. However, relatively few studies have examined career motivations of married women by focusing on individual differences and there have been limitations in developing and verifying research or theories to find mediating and moderating variables to help women continue their careers in the fields of counseling and education. The results of this study suggest that, beyond these limitations, the assumptions and theoretical framework of self-determination can be applied in explaining the career motivation of highly educated married women in collectivist cultures. Follow-up studies should examine the relationship between career persistence and career achievement and satisfaction and subjective psychological well-being in highly educated married women based on the SDT. In addition, further studies should verify the applicability of a program that can improve intrinsic motivation.

### Limitations and Future Research Directions

Several limitations of this research should be noted. First, the findings are based on a relatively small number of participants; thus, they might not accurately reflect the perspectives of the broader population of highly educated married women. In this research, the average age of participants was 35.03 years and 62.01% have one child. Thus, the participants of this study are relatively young and have few children. In addition, the participants in this study were highly educated women with bachelor’s and master’s degrees, and there were many people working in office jobs rather than professional jobs. Therefore, the results of this study are limited in applying to highly educated women with doctoral degrees or professional positions. Therefore, the research results should be further tested to improve external validity among a larger more representative sample of highly educated married women. Second, this research is based on participants’ self-reports. Retrospective recall can sometimes result in forgetting important aspects or nuances of experiences, because people recreate the memory based on other memories. Furthermore, this may occur because of the social demands of the interview situation (Nisbett and Wilson, 1977).

## Conclusion

The present study is the first to conceptualize career motivations of highly educated married women. Motivation is a psychological concept explored by various theories, and vigorous research is being conducted to test these theories in the field of career research using motivation as the key concept. Therefore, this study conceptualized the motives of the highly educated married women and laid the foundation for subsequent studies. In particular, the lack of research on the career development and choices of highly educated married women in the context of psychological theories and concepts has consistently been pointed out. The present study expects to somewhat ameliorate this deficiency and provide a starting point to future research.

## Data Availability Statement

The datasets generated for this study are available on request to the corresponding author.

## Ethics Statement

Ethical review and approval was not required for the study on human participants in accordance with the local legislation and institutional requirements. The patients/participants provided their written informed consent to participate in this study.

## Author Contributions

MK contributed to conception and design of the study, organized the database, performed the statistical analysis, wrote the first draft of the manuscript, and wrote sections of the manuscript.

## Conflict of Interest

The author declares that the research was conducted in the absence of any commercial or financial relationships that could be construed as a potential conflict of interest.

## Footnotes

1 Career interruption can be defined as a situation in which an individual has stopped work or is not temporarily working.

## References

[B1] Ashmore R. D. Del Boca F. K. Wohlers A. J. (1986). “Gender stereotypes,” in *The Social Psychology of Female-Male Relations*, eds Ashmore R. D. Del Boca F. K. (Orlando, FL: Academic Press).

[B2] Ashmore R. D. Tumia M. L. (1980). Sex stereotypes and implicit personality theory: a personality description approach to the assessment of sex stereotypes. *Sex Roles* 6 501–518.

[B3] Baard P. P. Deci E. L. Ryan R. M. (2004). Intrinsic need satisfaction: a motivational basis of performance and well-being in two work settings. *J. Appl. Soc. Psychol.* 34 2045–2068.

[B4] Bae K. Lee D. Hwang M. H. Lee S. Cha J. Jeong H. (2015). Cross-cultural comparison between Korean and Japanese mother’s career guidance. *Asian J. Educ.* 16 83–115.

[B5] Becker G. S. (1991). *A Treatise on the Family.* Cambridge: Harvard UniversityPress.

[B6] Bedi R. P. Alexander D. A. (2009). Using multivariate concept-mapping for examining client understandings of counselling. *Can. J. Counsell.* 43 76–91.

[B7] Bem S. L. (1993). *The Lenses of Gender: Transforming the Debate on Sexual Inequality.* New Haven, CT: Yale University Press.

[B8] Bezzina F. Azzopardi R. M. Vella G. (2013). Understanding and assessing the work motivations of employed women: insights into increasing female participation rates in the maltese labor market. *Sage Open* 3 1–14.

[B9] Blais M. R. Lachance L. Vallerand R. J. Brie‘re N. M. Riddle A. S. (1993). The Blais Inventory of Work Motivation [French]. *Rev. Que’-be’coise Psychol.* 14 185–215.

[B10] Cho S. I. (2009). *Research on Reentry of Women with Interrupted Work Career to Labor Market-Focused on Highly Educated Women*, (Unpublished master’s thesis)., Yonsei University, Seoul.

[B11] Choi E. Y. (2016). A study on married women‘s participation in economic activity and number of children. *Women’s Econ. Res.* 31 51–70.

[B12] Choi H. M. (2014). *Labor Market Participation of Married Women and the Dependency of Low-Paid Labor.* Sejong-si: Korean Labor Institute.

[B13] Choi H. M. Yoo H. M. Kim J. H. Kim T. W. (2016). *Awareness of the Non-Marriage of Young People and Countermeasures Against Low Birth Rate.* Seoul: Korea Institute of Childcare and Education.

[B14] Choi H. S. (2017). *Recent Growth in Women’s Professions and Labor Market Performance.* Seoul: Korea Institute for Industrial Economics and Trade.

[B15] Choi Y. J. Kim K. H. (2007). Concept mapping the highly educated and married women’s experience on crisis of career interruption for comparing career women with housewives had experienced career interruption. *Korea J. Counsel.* 8 1031–1045.

[B16] Clarke K. R. (1993). Non-parametric multivariate analyses of changes in community structure. *Austral Ecol.* 18 117–143.

[B17] Clayton D. E. Smith M. M. (1987). Motivational typology of reentry women. *Adult Educ. Quart.* 37 90–104.

[B18] Crompton R. (2001). Gender restructuring, empolyment and caring. *Soc. Politcs* 8 266–291.

[B19] Cury F. Elliot A. J. Da Fonseca D. Moller A. C. (2006). The social-cognitive model of achievement motivation and the 2 × 2 achievement goal framework. *J. Pers. Soc. Psychol.* 90 666–679. 16649862 10.1037/0022-3514.90.4.666

[B20] Daughtry D. Kunkel M. A. (1993). Experience of depression in college students: a concept map. *J. Counsel. Psychol.* 40 316–323.

[B21] Deci E. L. (1971). Effects of externally mediated rewards on intrinsic motivation. *J. Pers. Soc. Psychol.* 18 105–115.

[B22] Deci E. L. (1975). *Intrinsic Motivation.* New York, NY: Plenum.

[B23] Deci E. L. Ryan R. M. (2000). The ‘what’ and ‘why’ of goal pursuit: human needs and the self-determination of behavior. *Psychol. Inq.* 11 227–268.

[B24] Del Boca D. Pasqua S. Pronzato C. (2009). Motherhood and market work decisions in institutional context: a european perspective. *Oxf. Econ. Papers* 61(Suppl. 1), i147–i171.

[B25] Desai S. Waite L. (1991). Women’s employment during pregnancy and after the first birth : occupational characteristics and work commitment. *Am. Sociol. Rev.* 56 551–566.

[B26] Dubey N. Tiwari V. (2014). Factors affecting career aspiration among married women. *Ind. J. Health Wellbe.* 5 59–63.

[B27] Elliot A. J. (1999). Approach and avoidance motivation and achievement goals. *Educ. Psychol.* 34 149–169.

[B28] Elvin-Nowak Y. Thomsson H. (2001). Motherhood as idea and practice: a discursive understanding of employed mothers in Sweden. *Gender Soc.* 15 407–428.

[B29] Everitt B. S. Landau S. Leese M. Stahl D. (2011). *Cluster Analysis.* Chichester: John Wiley and Sons Ltd.

[B30] Faisal F. Khan M. M. (2011). Individual and structural determinants of intrinsic work preference among female public servants in Pakistan. *Austr. J. Bus. Manage. Res.* 1 63–74.

[B31] Fernet C. Guay F. Senécal C. (2004). Adjusting to job demands: the role of work self-determination and job control in predicting burnout. *J. Vocat. Behav.* 65 39–56.

[B32] Gagné M. Chemolli E. Forest J. Koestner R. (2008). A temporal analysis of the relation between organisational commitment and work motivation. *Psychol. Belgica* 48 219–241.

[B33] Geist C. (2005). The welfare state and the home: regime differences in the domestic division of labour. *Eur. Sociol. Rev.* 21 23–41.

[B34] Goldin C. (2006). The quiet revolution that transformed women’s employment, education, and family. *Am. Econ. Rev.* 96 1–21.

[B35] Hair J. F. Black W. C. (2000). “Cluster analysis,” in *Reading and Understanding More Multivariate Statistics*, eds Grimm L. G. Yarnold P. R. (Washington, DC: American Psychological Association), 147–205.

[B36] Harter J. K. Schmidt F. L. Hayes T. L. (2002). Business-unit-level relationship between employee satisfaction, employee engagement, and business outcomes: a meta-analysis. *J. Appl. Psychol.* 87 268–279. 12002955 10.1037/0021-9010.87.2.268

[B37] Heiss J. (1981). “Social roles,” in *Social Psychology Sociological Perspectives*, eds Rosenberg M. Tuner R. H. (New York: Basic Books Inc), 94–129.

[B38] Hong S. A. Lee I. (2014). *Father’s Use of Parental Leave in Korea: Motives, Experience and Problems.* Seoul: Korean Women’s Development Institute.

[B39] Houkes J. Jansen P. De Jonge J. Nijhuis F. (2001). Work and individual determinants of intrinsic work motivation, emotional exhaustion, and turnover intention: multi-sample analyses international. *J. Stress Manage.* 8 257–283.

[B40] Ismail H. C. (2012). An exploratory study of motivational factors on women entrepreneurship venturing in Malaysia. *Bus. Econ. Res.* 2 282–297.

[B41] Jang J. Y. Bu G. C. (2003). *Hidden Choice: Paid work and Child Care for Married Female Workers.* Seoul: Korean Women’s Development Institute.

[B42] Jang S. Merriam S. (2004). Korean culture and the reentry motivations of university graduated women. *Adult Educ. Q.* 54 273–290. 10.1177/0741713604266140

[B43] Jang S. Y. (2002). *How University Attendance Affects the Personal Development of Highly Educated Middle Class Korean Reentry Women.* PhD dissertation University of Georgia, Athens, GR.

[B44] Jang S. Y. (2008). The qualitative research on the reentry process of highly educated housewife to their job market. *Women’s Stud.* 74 79–104.

[B45] Jang S. Y. Merriam S. (2005). Korean culture and the reentry motivations of university women. *Adult Educ. Quart.* 54 23–51.

[B46] Jeon H. S. (2010). *A Study on Learning Experiences of Woman Who Returned to Work After Career Breaks: A ground Theory.* (Unpublished doctoral dissertation)., Busan National University, Busan

[B47] Jeon Y. S. Ju Y. A. (2016). A consensual qualitative research on experiencing help-seeking behavior of career interrupted women. *J. Employment Career* 6 45–70.

[B48] Johnston D. D. Swanson D. H. (2006). Constructing the “god mother”: the experience of mothering ideologies by work status. *Sex Roles* 54 509–519.

[B49] Joo H. J. (2005). Effects of gender stereotype on the evaluation of women managers. *Korean Assoc. Women’s Stud.* 22 115–149.

[B50] Ju S. H. Joo Y. K. (2017). A study on career preparation process from middle-aged women’s career break to study participation. *Korean Soc. Fish. Sci.* 29 1446–1459.

[B51] Jung J. M. Kim H. Y. (2015). *Development of Work and Home Compatibility Support Model for Double-Income Families.* Seoul: Ministry of Gender Equality and Family.

[B52] Jung M. H. (2012). *A Qualitative Study on Milled-Aged Married Women’s Job Career Continuance Process.* (Unpublished doctoral dissertation)., Honam University, Gwangju.

[B53] Kane M. Trochim W. M. K. (2007). *Concept Mapping for Planning and Evaluation.* Thousand Oaks, CA: Sage.

[B54] Kang L. S. (2011). The changes of husband’s attitudes to work-family problems in dual earner families. *Gender Cult.* 4 43–87.

[B55] Kasser T. Ryan R. M. (1993). A dark side of the American dream: correlates of financial success as a central life aspiration. *J. Pers. Soc. Psychol.* 65 410–422. 8366427 10.1037//0022-3514.65.2.410

[B56] Kasser T. Ryan R. M. (1996). Further examining the American dream: differential correlates of intrinsic and extrinsic goals. *Personal. Soc. Psychol. Bull.* 22 80–87.

[B57] Kim B. S. (2014). Job change of highly educated married women (aged 35–39). *Monthly Lab. Rev.* 3 97–99.

[B58] Kim E. J. Seo Y. H. (2012). A study on child rearing experience of stay at home mon with infants. *Early Childhood Educ. Care* 9 93–114.

[B59] Kim H. O. Siegfried H. D. (2001). Mothers roles in traditional and modern Korean families: the consequence for parental practices and adolescent socialization. *Asia Pacific Educ. Rev.* 2 85–93.

[B60] Kim H. Y. Sun B. Y. Kim S. D. (2010). *Women’s Late Marriage and Low Fertility.* Seoul: Korean Women’s Development Institute.

[B61] Kim J. K. (2003). *Analysis of Married Women’s Career Breaks and Return to Labor Market After Giving Birth.* Sejong-si: Korean Labor Institute.

[B62] Kim M. S. Seo Y. S. (2015). Clustering by career persistence motivations and group differences in work burnout and life satisfaction among highly educated married working women. *Korean J. Counsel. Psychol.* 27 425–443.

[B63] Kim N. J. (2016). Analysis on career-interrupted women’s reentry to labor market and maintaining the reemployment. *Korean J. Ind. Relat* 26 1–27.

[B64] Kim S. H. Park J. Y. (2012). The relationship between personality and child rearing stress of employed mothers. *J. Korean Home Econ. Assoc.* 50 41–52.

[B65] Kim Y. K. (2007). Need analysis for the career building and reemployment plans of career interrupted highly educated women. *Women’s Stud.* 73 85–118.

[B66] Kochhar K. Jain-Chandra S. Newiak M. (eds) (2016). *Women, Work, and Economic Growth: Leveling the Playing Field.* Washington, DC: International Monetary Fund.

[B67] Korea Research Institute for Vocational Education Training (2018). *KRIVET Issue Brief, Wage gap by nature using position information divided by difference and discrimination.* Daejeon: Korea Research Institute for Vocational Education Training.

[B68] Kruskal J. B. Wish M. (1978). *Multidimensional scaling.* Newbury Park, CA: Sage.

[B69] Kuvaas B. (2009). A test of hypotheses derived from self-determination theory among public sector employees. *Employee Relat.* 31 39–56.

[B70] Lee H. J. Kim K. M. (2010). The effects of perceived career barrier of women whose career were interrupted on career preparation behavior. *Korean J. Counsel.* 11 623–640.

[B71] Lee H. S. (2003). *Study on Interruption and Maintenance in Married Women’s Work Duration: Through Female Police Officers’ Work Experience.* (Unpublished doctoral dissertation)., Seoul: Ewha Women’s University.

[B72] Lee H. W. (2014). Changes in the labor supply of married women by marriage and birth. *Monthly Public Finance Forum* 216 39–58.

[B73] Lee S. B. Lee J. H. (2010). The influence of career barriers perceived by unemployment married women on career preparation behaviors. *J. Vocat. Educ. Res.* 29 187–208.

[B74] Lee S. B. Lee J. H. (2014). A qualitative research on the career choice of married women for employment preparation. *J. Hum. Resour. Manage. Res.* 12 165–185.

[B75] Lee Y. J. Kim E. J. (2014). Dynamic analysis on re-employment of married women: focusing on high educated women. *J. Korean Natl. Econ.* 32 87–114.

[B76] Lewis J. (2001). The decline of the male-breadwiner model: implications for work and care. *Soc. Policy* 2 159–173.

[B77] Milette V. Gagné M. (2008). Designing volunteers’ tasks to maximize motivation, satisfaction and performance: the impact of job characteristics on the outcomes of volunteer involvement. *Motiv. Emot.* 32 11–22.

[B78] Ministry of Gender Equality and Family (2013). *The Life of Women in Statistics.* Seoul: Korea.

[B79] Nisbett R. E. Wilson T. D. (1977). Telling more than we can know: verbal reports on mental processes. *Psychol. Rev.* 84 231–257.

[B80] OECD (2017). *The Pursuit of Gender Equality: An Uphill Battle.* Paris: OECD Publishing.

[B81] Otis N. Pelletier L. G. (2005). A motivational model of daily hassles, physical symptoms, and future work intentions among police officers. *J. Appl. Soc. Psychol.* 35 2193–2214.

[B82] Paulson B. L. Truscott D. Stuart J. (1999). Clients’ perceptions of helpful experiences in counseling. *J. Counsel. Psychol.* 46 317–324.

[B83] Pinder C. C. (1998). *Work Motivation: Theory, Issues, and Applications.* Glenview Illinois: Scott, Foreman and Company.

[B84] Powell G. N. Butterfield D. A. Parent J. D. (2002). Gender and managerial stereotypes: have the times changed? *J. Manage.* 28 177–193.

[B85] Ratelle C. F. Larose S. Guay F. Senecal C. (2005). Perceptions ofparental involvement and support as predictors of college students’persistence in a science curriculum. *J. Fam. Psychol.* 19 286–293. 15982106 10.1037/0893-3200.19.2.286

[B86] Richer S. F. Blanchard C. Vallerand R. J. (2002). A motivational model of work turnover. *J. Appl. Soc. Psychol.* 32 2089–2113.

[B87] Rosada I. Sukesi K. Sugiyanto Yuliati Y. (2013). Characteristics and motivating factors for the double role of informal female workers: a study of dange Vendors in the village of bodie, district of mandalle, regency of pangkep, province of south sulawesi. *J. Basic Appl. Sci. Res.* 3 226–236.

[B88] Ryan R. M. Deci E. L. (2000). Self-determination theory and the facilitation of intrinsic motivation, social development, and well-being. *Am. Psychol.* 55 68–78. 10.1037/0003-066X.55.1.68 11392867

[B89] Ryu J. Y. Kim S. R. Lee S. L. (2016). A qualitative study on the reemployment trial of career interrupted women in their 30s. *J. Learn. Center. Curric. Instr.* 16 931–964.

[B90] Son J. H. (2013). Career experiences of highly educated professional women focusing on women with 5-10 career years. *Korean J. Counsel.* 14 501–522.

[B91] Statistics Korea (2015). *Work and Family Compatibility Indicators 2015.* Available online at: http://kostat.go.kr (accessed 5 June, 2018).

[B92] Statistics Korea (2016). *Women’s Life in Statistics 2016.* Available online at: http://kostat.go.kr, (accessed 5 June, 2018)

[B93] Sung M. A. Ok S. H. (2006). A qualitative study of retirees from the teaching and public service professions: career sustaining strategies of female retirees in South Korea. *J. Korean Home Manage.* 24 67–87.

[B94] Trochim W. M. K. (1989). An introduction to concept mapping for planning and evaluation. *Eval. Program Plann.* 12 1–16.

[B95] Trochim W. M. K. (1993). The reliability of concept mapping. *Paper presented at the Annual Conference of the American Evaluation Association*, Dallas, TX.

[B96] Trochim W. M. K. Cook J. A. Setze R. J. (1994). Using concept mapping to develop a conceptual framework of staff’s views of a supported employment program for individuals with severe mental illness. *J. Consult. Clin. Psychol.* 62 766–775. 7962880 10.1037//0022-006x.62.4.766

[B97] Um K. A. (2010). *A Qualitative Study on the Career Interrupted and the Child Care of Highly Educated Housewives.* (Unpublished doctoral dissertation)., Injae University, Jeon Nam.

[B98] Um K. A. Yang S. E. (2011). A qualitative study on the career interrupted and the child care of married women. *J. Korean Home Manage. Assoc.* 29 21–40.

[B99] Wheeldon J. (2010). Mapping mixed methods research: methods, measures, and meaning. *J. Mixed Methods Res.* 4 87–102.

[B100] White K. S. Farrell A. D. (2001). Structure of anxiety symptoms in urban children: competing factor models of the revised children’s manifest anxiety scale. *J. Consult. Clin. Psychol.* 69 333–337. 11393610

[B101] Won S. Y. (2014). The implications of gender-role fixation on the topography and women’s policy: focusing on the perception differences between 1996 and 2010. *Govern. Stud.* 20 141–171.

[B102] Won S. Y. (2015). Multidimensionality and antecedents of job satisfaction perceived by working mothers: a focus on perceived discrimination and wokr-family support. *Korean J. Public Admin.* 53 57–81.

[B103] Yang S. N. Shin C. S. (2011). Working family conflicts challenges of working mothers with young children. *Health Soc. Welf. Rev.* 31 10–103.

[B104] Yang S. Y. (2018). A Study on the Problems of Women’s Career Interruption and Overseas Cases. Seoul: KDB Research.

[B105] Yoo H. M. Bae Y. J. Kim M. J. (2014). *A Study on the Current Status and Improvement of Child Care Support for Dual-Income Families.* Seoul: Korea Institute of Childcare and Education.

[B106] Yoo S. K. Lim J. S. Son E. Y. (2016). Concept mapping of work-family reconciliation in Korean working mothers. *Korean J. Counsel.* 17 475–498.

[B107] Yoon H. K. (2007). *An Analysis on Vocational Exploration Experiences of Highly Educated Women with Discontinued Employment: With a Focus on the Middle-Aged Participants in the Vocation Education Program.* (Unpublished Doctoral Dissertation)., Ewha Womans University, Seoul.

[B108] Yu M. M. (2005). “Domestic violence in the Chinese American community,” in *Domestic Violence in Asian American Communities*, ed. Nguyeon T. D. (Lanham, MD: Lexington Books), 27–38.

[B109] Zusho A. Pintrich P. R. Coppola B. (2003). Skill and will: the role of motivation and cognition in the learning of college chemistry. *Int. J. Sci. Educ.* 25 1081–1094.

